# South Africa

**Published:** 1897-01

**Authors:** 


*South Africa.* Rinderpest continues to prevail in South Africa, and as usual remedies of every conceivable kind are springing up. The disease is in some measure controlled by sanitary police-system, but the difficulties in maintaining this rigid enough are many and hard to deal with, as the natives are not of sufficient intelligence to appreciate the value of these measures.

